# Physiological Effects of Water Flow Induced Swimming Exercise in Seabream *Sparus aurata*

**DOI:** 10.3389/fphys.2020.610049

**Published:** 2020-12-07

**Authors:** Arjan P. Palstra, Ana Roque, Leo Kruijt, Pauline Jéhannet, Jaume Pérez-Sánchez, Ron P. Dirks

**Affiliations:** ^1^Wageningen University & Research Animal Breeding and Genomics, Wageningen Livestock Research, Wageningen, Netherlands; ^2^IRTA-SCR, Sant Carles de la Rapita, Spain; ^3^Nutrigenomics and Fish Growth Endocrinology Group, Institute of Aquaculture Torre de la Sal (CSIC), Castellon, Spain; ^4^Future Genomics Technologies B.V., Leiden, Netherlands

**Keywords:** aquaculture, robustness, growth performance, stress resilience, vertebral lordosis, RNAseq

## Abstract

A longer on-land rearing period of Gilthead seabream *Sparus aurata* before transfer to sea-cages would allow the farmer to benefit from exercise-enhanced growth, resilience, and robustness as induced by increasing water flow in the tanks. In this study, the physiological effects of flow-conditioning were investigated by subjecting large groups of experimental fish to minimal flow or to flow regimes inducing swimming exercise at 1 or 2 body length (BL) s^−1^ for a period of 8 months (February–October) in 1,500 L tanks. Fish representing the three treatment groups were then used for: (1) a stress challenge netting test and plasma cortisol measurement (baseline, peaking, and recovery levels), (2) blood plasma measurements of glucose, triglycerides, lactate, cholesterol, growth hormone (GH), and insulin-like growth factor 1 (IGF1), and (3) heart and muscle gene expression of the GH and IGF1 receptors and the muscle transcriptome by deep RNA sequencing (RNAseq). Fish size after 8 months of flow conditioning was 92 ± 27 g body weight (BW) for fish under minimal flow, 106 ± 24 g BW (+15%) at 1 BL s^−1^, and 125 ± 27 g BW (+36%) at 2 BL s^−1^. Flow conditioning at 1 BL s^−1^ provided optimal conditions for growth and uniformity, but also stress (lowest baseline plasma cortisol), robustness (higher condition factor and larger hearts), and energy mobilization (increased plasma glucose). Although flow enhanced growth linearly with swimming speed, also the percentage of lordotic fish increased with exercise, particularly high for swimming at 2 BL s^−1^. The absence of important differences in plasma GH and IGF1, and expression levels of their receptors in heart and white skeletal muscle, indicated that other factors may be involved in growth enhancement. RNAseq of the white skeletal muscle showed upregulated expression of genes involved in muscle contraction, muscle development and its molecular regulation, and immune genes that may play a role in the muscle repair mechanism. An exercise regime of swimming at 1 BL s^−1^ can be considered as optimal for farming robust seabream although the increase of skeletal deformities should be avoided.

## Introduction

Gilthead seabream (*Sparus aurata*) is currently the most important species in Mediterranean aquaculture. Seabream is raised on-land in recirculating or flow through systems until sizes of 2 up to 20 g when they are transferred to sea-cages. During the on-land period, juvenile fish can be conditioned by swimming against the flow in order to stimulate (muscle) growth and increase robustness (Davison, 1997; Palstra and Planas, 2011; McKenzie et al., 2020). By holding fish on-land up to a size of 20 g or even larger under an increased flow regime, the farmer could benefit from increased robustness as mitigation measure against the negative physiological impact of handling, transportation, and acclimation to a novel environment, all associated with transfer to sea.

In case of seabream fingerlings and juveniles, long-term exercise significantly enhances growth performance (Ibarz et al., 2011; Sánchez-Gurmaches et al., 2013; Blasco et al., 2015; Vélez et al., 2016). The fingerlings of this species in the range of 5 up to 20 g increased 18% more in body weight than the controls when exercised at ~0.25 m s^−1^ for a 5-week period, corresponding to 5 BL s^−1^ at the start of the experimental period and decreasing to ~2.8 BL s^−1^ at the end (Blasco et al., 2015). The metabolic optimal swimming speed is the speed where fish swim most efficiently and which is linked to optimal growth for fish with high metabolic scope (Davison and Herbert, 2013). For juvenile seabream, highest exercise-enhanced growth rates are not found at the metabolic optimal swimming speed (4.51 ± 0.99 Sl s^−1^ for fish of 68 ± 21 g; Palstra et al., 2020; unpublished swim-training data, Graziano and Palstra), but at much lower swimming speeds. Ibarz et al. (2011) reported a 9% increase in body weight for seabream juveniles of ~90 g at the start that were exercised at 1.5 BL s^−1^ for 1 month. A major role for the growth hormone (GH)/insulin-like growth factors (IGFs) axis is expected in the endocrine regulation of exercised-enhanced growth, also in seabream. Hepatic IGF1 is produced and released into the circulation of trained seabream, where the IGF1/GH ratio rises (in fingerlings and juveniles; Sánchez-Gurmaches et al., 2013; Blasco et al., 2015; Vélez et al., 2016). In parallel with the increased IGF1/GH ratio, gene expression of several members of the GH-IGFs axis in the muscle differentiates, suggesting a functional relation in fingerlings (Vélez et al., 2016). An exercise-induced protein-sparing effect allows for the muscle building in juvenile seabream (Felip et al., 2013).

Long-term swimming exercise can be considered as physical stressor that impacts secretion of cortisol, either lowering plasma levels in salmonids (Boesgaard et al., 1993; Herbert et al., 2011) or increasing them in zebrafish (Palstra et al., 2019). However, cortisol signaling through the glucocorticoid receptor does not seem to play an important role in exercise-enhanced growth of zebrafish (Palstra et al., 2019). Exercise training may also modulate the cortisol stress response (Young and Cech, 1994), thereby potentially lowering the magnitude of the peak levels and/or shortening recovery times (Veiseth et al., 2006). As such, the cortisol stress response reflects the fish’ resilience or “the capacity of the animal to be minimally affected by a disturbance or to rapidly return to homeostasis” (Colditz and Hine, 2016).

Besides exercise effects on growth and stress coping, body changes occur as training adaptation reflecting a more aerobic phenotype. A more hydrodynamic and leaner body shape (Koumoundouros et al., 2009) with less body cavity volume available for tissues (Palstra et al., 2020), a larger heart with higher pumping capacity and cardiac output (Farrell, 1991; Farrell et al., 2007, 2009), changes in plasma levels of fuels for muscle contraction (Magnoni et al., 2013), and increased muscle angiogenesis (Palstra et al., 2014; Moya et al., 2019) can be expected. The mechanical load on the skeleton that exercise brings changes developmental prioritization (Fiaz et al., 2012) and leads to more skeletal mass (Totland et al., 2011; Suniaga et al., 2018). Such body changes may contribute to the fish’ robustness or “the capacity to maintain productivity in a wide range of environments without compromising reproduction, health and wellbeing” (Knap, 2005; Colditz and Hine, 2016).

In the present study, we have assessed the physiological effects of long-term water flow induced swimming exercise in juvenile seabream at 1 and 2 BL s^−1^ for an 8-month period vs. controls at minimal flow. The effects on growth, the cortisol stress response, biometry and tissue weights, energy metabolism, growth axis parameters, and the white skeletal muscle transcriptome were assessed in order to grasp the full physiological array of exercise effects and gain mechanistic insights in the molecular regulation of muscle development in juvenile seabream. Conclusions will contribute to provide advice to farmers on how to benefit from water flow induced swimming exercise to increase growth and robustness of seabream during an extended on-land rearing period.

## Materials and Methods

### Experimental Set-Up: Flow Conditioning and Sampling

Experiments were conducted at IRTA facilities (Sant Carles de la Ràpita, Spain). Three 1,500 L conic tanks (150 cm diameter), connected in a recirculating aquaculture system, were modified for flow-conditioning: inner PVC rings of 20 cm diameter with holes on the lower side for self-cleaning were placed over the center drainage, water level was reduced to half, and water inlet came through flattened pipes. Flow velocity measurements were performed with a FP101 water flow probe and showed that flow was homogenous and maximally 0.51 m s^−1^. Flow velocity was linked to a valve position in order to adjust in relation to fish size. At the end of January, *N* = ~3,300 fish (BW ~ 2 g; BL 39 ± 4 mm on basis of measuring 30 fish) were purchased from Culmar (Alicante, Spain) and randomly divided over the tanks (so ~1,100 fish per tank with a density of 3 kg m^−3^ that was maintained by monthly random removal of fish).

After an acclimation period of 2 weeks, one tank was kept at minimal flow while flow conditioning in the two other tanks commenced with swimming at 1 and 2 BL s^−1^, respectively [with standard length (SL) taken as body length (BL)]. At every feeding event, flow was stopped for 20 min. Fish were manually fed twice per day *ad libitum* with commercial seabream pellets (Skretting, Spain). As fish grew bigger, pellet size was adjusted by mixing the 2 sizes for a week before switching to the larger size.

Water temperature and dissolved oxygen (DO) were automatically measured every 5 min. Water temperature was 18 ± 0.5°C and DO 6.0 ± 0.5 mg L^−1^ over the whole experimental period. Salinity was kept at 35 ppt, and the light regime was natural. pH was measured weekly with a handheld probe (YSI) and was in the range of 7.1–7.8. Ammonium and nitrite were measured once a week with Merck kits. Ammonium was considered zero throughout the experiment except for July when it was 1.6 mg L^−1^ but after adding bicarbonate, levels slowly decreased until the end of September. Nitrite started at 1.50 mg L^−1^, then increased during the next month to 3.75 mg L^−1^, and then down to stabilize at levels of 0.2–0.3 mg L^−1^.

Every 2 weeks during the first 2 months and subsequently every month (*N* = 11 measurements incl. start) over the period January–September, a subsample of fish per tank (*N* = 15–100 per group with representative *N* per group to keep variation over time similar) was measured for SL and weighed for BW to monitor growth and to adjust the swimming speed by further opening the valve and increasing water inlet pressure. After 8 months in October, *N* = 40 fish per tank were used for measurements and sampling. Fish were randomly scooped out of the tanks alternating between tanks and euthanized with an overdose of MS222 (70 mg L^−1^). Fish were photographed for analysis of morphological abnormalities, measured for BW and SL, and the condition factor *K* was calculated. Heart and mesenteric fat were dissected and weighed, and the cardiac index (CI) and the mesenteric fat index (MFI) were calculated as:

$$\left(\mathrm{tissue weight}/\mathrm{BW}\right)*100\%$$

Of the first *N* = 10 of these *N* = 40 fish per tank, a blood sample (2 × 1 ml) was taken from the dorsal aorta with heparin flushed syringe, placed on ice, then spinned, and plasma was stored at −80°C. Intestine, liver, and spleen were dissected and weighed, and the intestinal index (II), hepatosomatic index (HSI), and spleen index (SI) were calculated as described above. The left overs were weighed as carcass weight, and the carcass index was calculated. Duplicate white muscle tissue samples and heart samples were taken, fixed in liquid nitrogen, and then stored at −80 °C at IRTA and WUR.

### Experimental Set-Up: Stress Challenge Test and Sampling

After the 8 months of flow conditioning, a stress challenge test was performed. The stress challenge consisted of 3 min of netting stress (Arends et al., 1999). Groups of *N* = 10 fish were subjected to the netting stressor, and then allowed to recover group-wise in 60 L boxes with aerated water. Fish were sampled for blood immediately and without netting stress for baseline cortisol levels (*N* = 10 fish per treatment), and then at 30 and 90 min after netting stress (each *N* = 10 fish per treatment), supposedly reflecting peaking and decreasing cortisol levels, respectively. For this purpose, fish were very shortly anesthetized, and then rapidly sampled for blood which was processed as described before.

### Blood Plasma Measurements

Plasma cortisol levels were determined using the Fish cortisol ELISA Kit of Cusabio. Plasma glucose (gluc), cholesterol (chol), and triglyceride (tg) levels were enzymatically determined (tg without glycerol correction). Lactate (lact) levels were determined using the lactate dehydrogenase method converting lactate to pyruvate. Photometric measurements of gluc, chol, tg, and lact were performed by the Utrecht University Veterinary Diagnostic Laboratory using the Clinical Chemistry Analyzer Olympus AU 680 (Beckman Coulter, Woerden, Netherlands). Plasma growth hormone (GH) was determined by a homologous gilthead seabream radioimmunoassay (RIA; Martínez-Barberá et al., 1995). Sensitivity and midrange (ED50) of the GH RIA assay were 0.15 and 1.8 ng/ml, respectively. Plasma IGFs were extracted by acid-ethanol cryoprecipitation (Shimizu et al., 2000), and the concentration of IGF1 was measured by a generic fish IGF1 RIA validated for Mediterranean perciform fish (Vega-Rubín de Celis et al., 2004). Sensitivity and midrange of the IGF1 RIA assay were 0.05 and 0.7–0.8 ng/ml, respectively. The IGF1/GH ratio was calculated.

### Heart and White Skeletal Muscle RT-PCR

Quantitative real-time PCR was performed as previously described (Jéhannet et al., 2019). Briefly, Total RNA was isolated from heart and muscle with Trizol Reagent (Invitrogen, CA, United States). Traces of DNA were digested with recombinant DNAse I (Ambion, CA, United States), and RNA was transcribed to cDNA with PrimeScript RT Reagent kit (Takara, Kusatsu, Japan). Quantitative real-time PCR was performed with SYBR Green Master Mix (Takara, Kusatsu, Japan), run on the QuantStudio™-5 real-time PCR system (ThermoFisher, Waltham, Massachusetts, United States). PCR conditions were 40 cycles of denaturation at 95°C for 5 min, annealing temperature at 55–60°C for 10 s, and extension at 72°C for 5 s. Melting curve analysis was performed to check for primer-dimers artifacts and reaction specificity. Primer sequences and annealing temperature are presented in Table 1. Primer efficiencies were determined by generating standard curves for the housekeeping and target genes. *R*^2^-values and efficiency for all standard curves were >0.99 and 100–110%, respectively. Data were expressed as fold change by using the 2^−ΔΔCT^ method (Livak and Schmittgen, 2001), whereby the treatment groups were each compared with the control group (e.g., 1 BL s^−1^ vs. C and 2 BL s^−1^ vs. C).

**Table 1:** Primers for each of the target genes used in real-time PCR (RT-PCR) analysis.

Target genes	Abv.	Accession number	Primer sequences	T°	Reference
Ribosomal protein S18	Rps18	AM490061.1	FW: GGGTGTTGGCAGACGTTAC RV: CTTCTGCCTGTTGAGGAACCA	60	Vieira et al., 2012
Growth hormone receptor 1	GHR1	AF438176	FW: ACCTGTCAGCCACCACATGA RV: TCGTGCAGATCTGGGTCGTA	60	Calduch-Giner et al., 2003
Growth hormone receptor 2	GHR2	AY573601	FW: GAGTGAACCCGGCCTGACAG RV: GCGGTGGTATCTGATTCATGGT	60	Saera-Vila et al., 2005
Insulin-like growth factor 1 receptor a	IGF1Ra	KT156846	FW: AGCATCAAAGACGAACTGG RV: CTCCTCGCTGTAGAAGAAGC	55	Azizi et al., 2016
Insulin-like growth factor 1 receptor b	IGF1Rb	KT156847	FW: GCTAATGCGAATGTGTTGG RV: CGTCCTTTATGCTGCTGATG	55	Azizi et al., 2016

FW, forward; RV, reverse; T°, annealing temperature.

### White Muscle RNAseq

RNA from white muscle tissue (*N* = 3 individuals per group; similar sized fish with normal morphology) was isolated using a miRNeasy kit (Qiagen). Illumina multiplexed RNAseq libraries were prepared from 0.5 μg total RNA using the Illumina TruSeq Stranded mRNA Library Prep according to the manufacturer’s instructions (Illumina Inc.). RNA concentrations measured with the Bio-analyzer ranged between 513 and 1,034 ng μl^−1^, and RIN values were generally 8–8.5. All RNAseq libraries were sequenced on an Illumina NovaSeq6000 sequencer as Illumina Paired-end 2 × 150 nt run (10 Mreads; 3 Gb) according to the manufacturer’s protocol. Image analysis and base calling were done by the Illumina pipeline. A total of 8 up till 12 million raw read counts were derived per sample. Quantitative analysis of the RNAseq data sets was performed by the alignment of reads against the Gilthead seabream (*S. aurata*) reference genome (https://www.ncbi.nlm.nih.gov/genome/11609?genome_assembly_id=389906) using TopHat (version 2.0.13; Trapnell et al., 2009) and 3–6 million (38.8–41.1%) of the RNAseq reads could be mapped. Reference alignment was done, and the resulting files were filtered using SAMtools (version 1.2 using htslib 1.2.1; Li et al., 2009) to exclude secondary alignment of reads. For statistical comparison of gene expression levels between groups, aligned fragments per predicted gene were counted from SAM alignment files using the Python package HTSeq (version 0.6.1p1; Anders et al., 2014). In order to make comparisons across samples possible, these fragment counts were corrected for the total amount of sequencing performed for each sample. As a correction scaling factor, we employed library size estimates determined using the R/Bioconductor (release 3.3.2) package DESeq (Anders and Huber, 2010). Read counts were normalized by dividing the raw counts obtained from HTSeq by its scale factor. Aligned reads were processed using DESeq, whereby the treatment groups were each compared with the control group. Raw RNAseq data (reads) have been submitted to NCBI’s Gene Expression Omnibus (GEO) as GSE151668 study.^1^

### Statistics

All measurements were taken blinded to the treatment allocation of each experimental group. Statistical analyses were performed with IBM SPSS 25 software. Normality of distribution was tested for the smallest group sizes of *N* = 15 with Kolmogorov–Smirnov tests. Growth differences between experimental groups were analyzed by one-way ANOVA with Tukey’s *post hoc* correction. Also, biometrical data, tissue weights, plasma metabolites, and hormones were analyzed this way except for the not normally distributed glucose data. Non-parametric Kruskal–Wallis tests were used to analyze glucose level, *K*, hematocrit, tissue weight indices, IGF1/GH ratios and gene expressions fold change data, and Mann–Whitney tests to estimate the pairwise differences. Additionally, ANCOVA was performed for log transformed values of HW and BW as cofactor. A value of *p* < 0.05 was considered significant.

Differentially expressed genes (DEGs) of each treatment group vs. control group were identified and analyzed, and treatment effects compared. Because of the high individual variation among fish in general, FDR correction was not applied at these limited numbers of fish per group. Instead, all identified DEGs were scanned for expression differences on individual level which showed obvious difference in expression between groups with over 80% of the DEGs showing different expression for all fishes of the treated group vs. all fishes of the controls. Gene ontology (GO) for upregulated DEGs of each comparison was manually performed on biological process level using Genecards. Most frequently occurring GO categories with the individual DEGs that contributed were considered.

## Results

### Swimming Performance

Water flow was applied up to a maximum of 0.37 m s^−1^ corresponding to a maximum average size when swimming at 2 BL s^−1^. Experimental fish displayed swimming behavior in all tanks: spontaneous and in multiple directions for the controls at minimal flow and sustained and unidirectional when induced to swim under flow treatments. For both water flows that induced swimming at 1 and 2 BL s^−1^, fish were in fact swimming harder than the flow. This indicates that the induced flows are low burdens for their endurance capacity. Not any experimental fish showed obvious signs of fatigue. Few mortality occurred during the experimental period (C: *N* = 44, 1 BL s^−1^: *N* = 31, 2 BL s^−1^: *N* = 12) and without obvious relation to the applied exercise regime.

### Growth Performance

**Figure 1:**
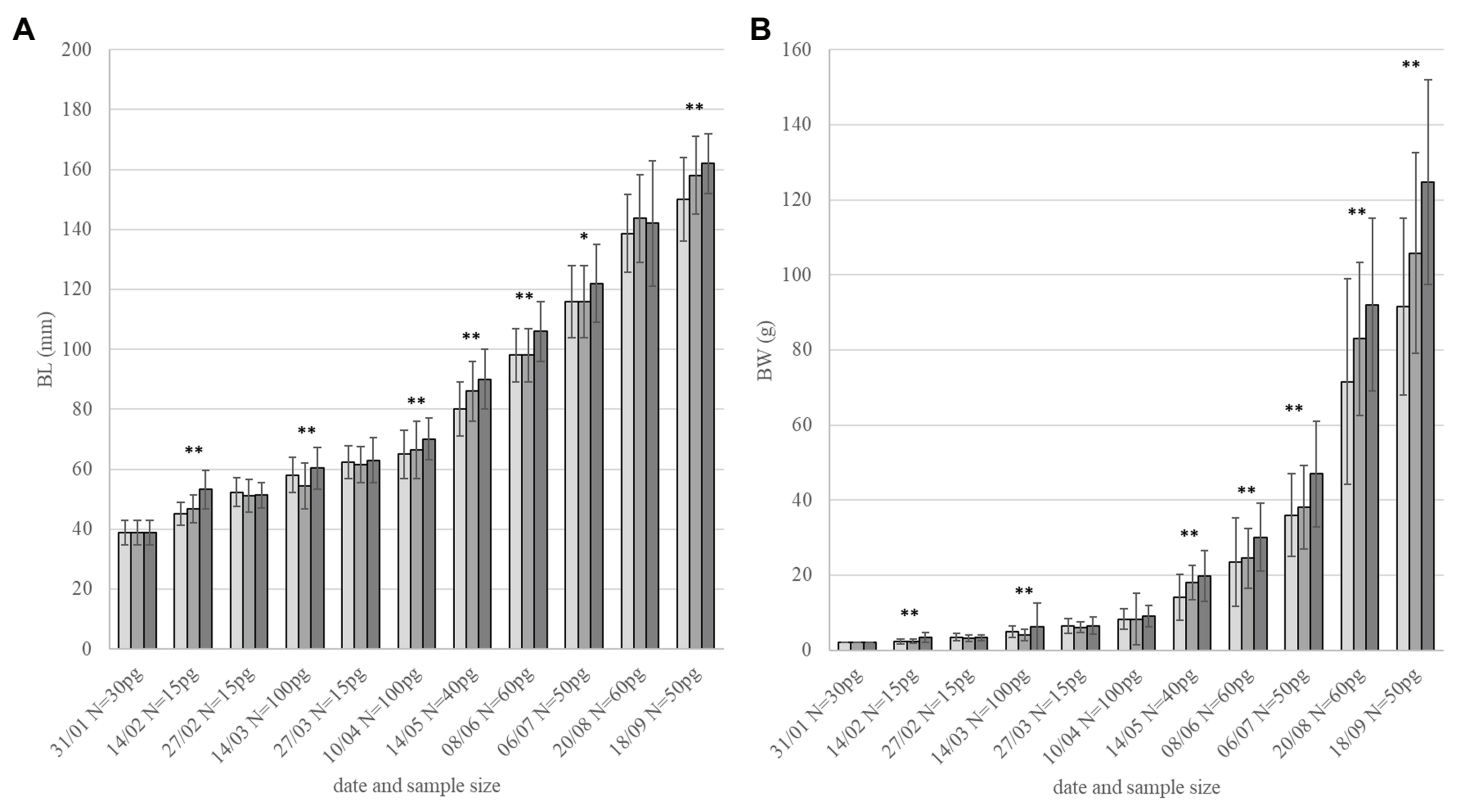
Growth performance. Fish size in **(A)** BL and **(B)** body weight (BW) over 8 months of flow conditioning (Jan-Sept, monitoring dates and sample size per group – pg. on the *x*-axis). Light gray: minimal flow. Medium gray: 1 BL s^−1^. Dark gray: 2 BL s^−1^. Asterisks indicate overall significant difference between groups for each date with ^*^p < 0.05 and ^**^p < 0.01.

During the whole experimental period, the order of experimental groups in growth rate was always similar (2 BL s^−1^ > 1 BL s^−1^ > minimal flow; Figure 1) but significance in differences was not always consistent. After 4 months, weight differences between groups were consistently significant (*p* < 0.001). Fish that were conditioned at 2 BL s^−1^ were significantly heavier than other groups (June 8th: *p* = 0.006 vs. 1 BL s^−1^ and *p* = 0.001 vs. controls; July 6th: *p* = 0.001 vs. 1 BL s^−1^ and controls). After 7 months, also fish conditioned at 1 BL s^−1^ were significantly heavier than the controls (August 20th: *p* = 0.023; September 18th: *p* = 0.019). Near to the end of the experimental period (September), body weight was 92 ± 27 g for fish at minimal flow; 106 ± 24 g (+15%) for fish at 1 BL s^−1^, and 125 ± 27 g (+36%) for fish at 2 BL s^−1^ (Figure 1). Growth was more uniform for the groups under flow (SD as percentage of average: 21.9 and 22.3%) than the group at minimal flow (29.2%).

### Biometry, Morphological Abnormalities, Tissue Weights, and Indices

Fish that were measured and dissected were of similar length but showed increase in weight in positive relation to the flow rate (*p* = 0.043) with fish conditioned at 2 BL s^−1^ being significantly heavier (188 ± 46 g vs. 177 ± 48 g and 162 ± 45 g for fish conditioned at 1 BL s^−1^ and the controls, respectively; *p* = 0.033; Table 2). As a result, *K* significantly increased in positive relation to the flow that was applied (*p* = 0.005).

**Table 2:** Morphology, biometry, tissue weight indices, hematocrit, plasma metabolites, and growth hormone growth hormone (GH) and insulin-like growth factor 1 (IGF1) levels.

		C AVG	SD	1 BL s^−1^ AVG	SD	2 BL s^−1^ AVG	SD
I.	lordosis	13%		23%		48%	
	SL (cm)	18.0	2.0	18.0	1.8	18.3	1.5
	BW (g)	**162**^**a**^	**45**	**177**^**ab**^	**48**	**188**^**b**^	**46**
	K	**2.72**^**a**^	**0.42**	**2.98**^**b**^	**0.39**	**3.07**^**c**^	**0.41**
	HW (g)	**0.19**^**a**^	**0.06**	**0.22**^**ab**^	**0.07**	**0.23**^**b**^	**0.07**
	CI	0.12	0.04	0.13	0.03	0.13	0.03
	MFW (g)	**1.69**^**a**^	**1.12**	**2.09**^**ab**^	**1.18**	**2.36**^**b**^	**1.01**
	MFI	**1.01**^**a**^	**0.56**	**1.14**^**ab**^	**0.47**	**1.25**^**b**^	**0.40**
II.	SW (g)	0.11	0.05	0.08	0.02	0.11	0.03
	SI	0.077	0.025	0.062	0.014	0.074	0.012
	LW (g)	1.48	0.80	1.39	0.34	1.51	0.43
	HSI	1.03	0.29	1.06	0.23	1.03	0.16
	IW (g)	3.70	0.98	3.63	0.54	4.12	0.79
	II	2.80	0.29	2.78	0.34	2.84	0.31
	carcass W (g)	120	36	113	14	131	29
	carcass I	89.4	0.8	85.5	10.4	89.8	0.9
	Hct %	38.5	5.8	40.1	4.6	36.7	3.8
	gluc (mmol L^−1^)	**3.9**^**a**^	**0.5**	**5.0**^**b**^	**0.9**	**3.9**^**a**^	**0.4**
	lact (mmol L^−1^)	4.0	1.1	4.8	1.6	3.4	1.2
	tg (mmol L^−1^)	3.2	0.9	2.9	0.6	2.6	0.7
	chol (mmol L^−1^)	7.2	0.9	7.1	0.5	7.1	0.9
	GH (ng ml^−1^)	13.84	8.88	10.59	6.85	6.23	2.92
	IGF1 (ng ml^−1^)	27.43	3.75	26.65	4.85	24.26	11.70
	IGF1/GH	1.74	0.56	3.71	2.43	4.21	3.53

Parameters (average AVG ± SD) in fish after conditioning for 8 months at minimal flow (C) and when induced to swim at 1 and 2 body length (BL) s^−1^. The first group of parameters (I) was monitored on a sample size of *N* = 40 fish per treatment group, the second group (II) on only the first *N* = 10 fish per treatment group. Significant differences (*p* < 0.05) are indicated in bold. For parameter abbreviations see text. ^a,^ ^b,^ ^c^ indicate what is similar and different from each other.

Photo analysis showed that abnormal morphology (e.g., vertebral lordosis) occurred at rates of 13 and 23% in control fish and fish conditioned at 1 BL s^−1^, respectively. In fish conditioned to swim at 2 BL s^−1^, the occurrence rate was much higher at 48%. In retrospect, first observation of lordosis was done on April 12th but must have been present already before that date.

Heart weight showed a significant difference between treatments (*p* = 0.004) with larger hearts in the fish that were conditioned to swim (*p* = 0.078 and *p* = 0.003 for fish swimming at 1 and 2 BL s^−1^). CI was not significantly higher in these fish, ANCOVA revealed a significant effect of the cofactor BW and a trend for HW (*p* = 0.087). Mesenteric fat also showed a significant difference between treatments (*p* = 0.028) with more fat in the fish that were conditioned to swim at 2 BL s^−1^ (*p* = 0.022). The MFI was also significantly higher in these fish (*p* = 0.007). Other tissue weights and indices did not reveal any significant differences between treatments.

### Hematocrit and Plasma Metabolites, GH and IGF1

Hematocrit and plasma glucose and lactate reflecting energy metabolism appeared to be higher in fish conditioned at 1 BL s^−1^, and then lower in fish conditioned at 2 BL s^−1^ vs. controls. This was only significant for glucose with 5.0 ± 0.9 vs. 3.9 ± 0.4 mmol L^−1^ in fish conditioned at 2 BL s^−1^ (*p* = 0.002) and 3.9 ± 0.5 mmol L^−1^ in the controls (*p* = 0.003). Triglycerides and cholesterol levels did not show any differences between treatment groups. Neither were plasma GH and IGF1 levels, and the IGF1/GH ratios, significantly different between treatment groups (Table 2).

### Stress Challenge Test and Plasma Cortisol

**Figure 2:**
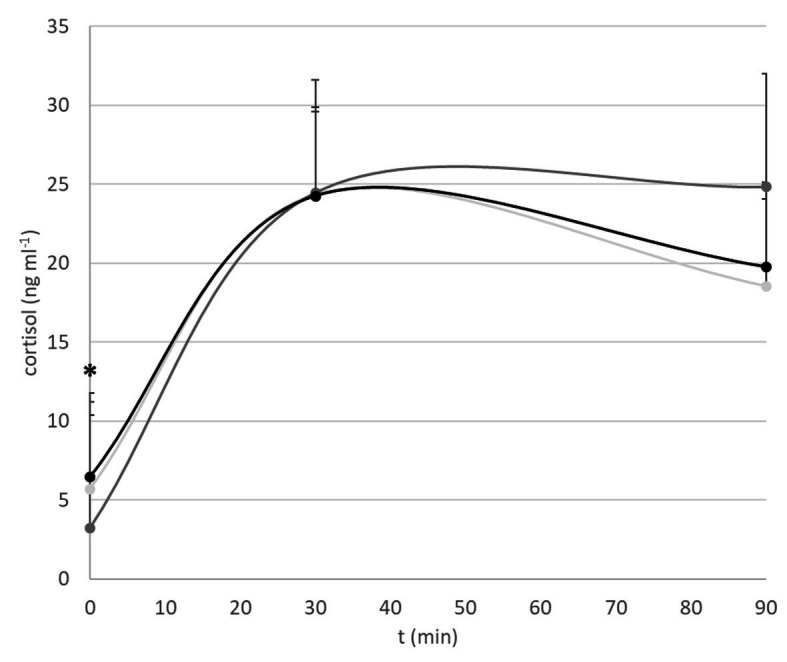
Cortisol stress response. Shown are baseline cortisol levels, and levels 30 and 90 min after netting stress. Light gray: minimal flow. Dark gray: 1 BL s^−1^. Black: 2 BL s^−1^. The asterisk indicates overall significant difference at ^*^p < 0.05: a lower baseline cortisol level of fish exercised at 1 BL s^−1^ vs. fish exercised at 2 BL s^−1^ and the controls.

Fish that were not subjected to netting stress revealed a significant difference in baseline cortisol levels (*p* = 0.04) with fish conditioned at 1 BL s^−1^ having lower levels (3.22 ± 1.28 ng ml^−1^) than fish conditioned at 2 BL s^−1^ (6.45 ± 3.03 ng ml^−1^; *p* = 0.05) or kept at minimal flow (5.72 ± 3.63 ng ml^−1^; ns *p* = 0.11; Figure 2). Thirty minutes after netting stress, cortisol levels were almost peaking and nearly identical between groups (C: 24.36 ± 6.76 ng ml^−1^; 1 BL s^−1^: 24.48 ± 4.77 ng ml^−1^; 2 BL s^−1^: 24.25 ± 10.18 ng ml^−1^). Ninety minutes after netting stress, no significant differences between treatment groups were apparent due to high individual variations (C: 18.57 ± 10.12 ng ml^−1^; 1 BL s^−1^: 24.85 ± 10.00 ng ml^−1^; 2 BL s^−1^: 19.77 ± 13.40 ng ml^−1^; *p* = 0.56).

### Heart and White Skeletal Muscle Gene Expression of the GH and IGF1 Receptors

No differential gene expression was found of *ghr1*, *ghr2*, *igf1ra^,^* and *igf1rb*, not in the heart and not in white muscle tissue. Fold changes were between 0.62 (white muscle *ghr2*) and 1.27 (heart *ghr1*) comparing exercise groups with the control.

### White Skeletal Muscle Transcriptome by RNAseq

The comparison 1 BL s^−1^ vs. controls yielded 13,211 transcripts that were associated with NCBI *S. aurata* genes (see Table S1). Around 97 genes were significantly expressed at *p* < 0.05. The expression of 44 genes was upregulated, and of 53 genes downregulated. The comparison 2 BL s^−1^ vs. controls yielded 13,487 transcripts that were associated with NCBI *S. aurata* genes (see Table S2). Around 75 genes were significantly expressed at *p* < 0.05. The expression of 43 genes was upregulated and of 32 genes downregulated.

**Figure 3:**
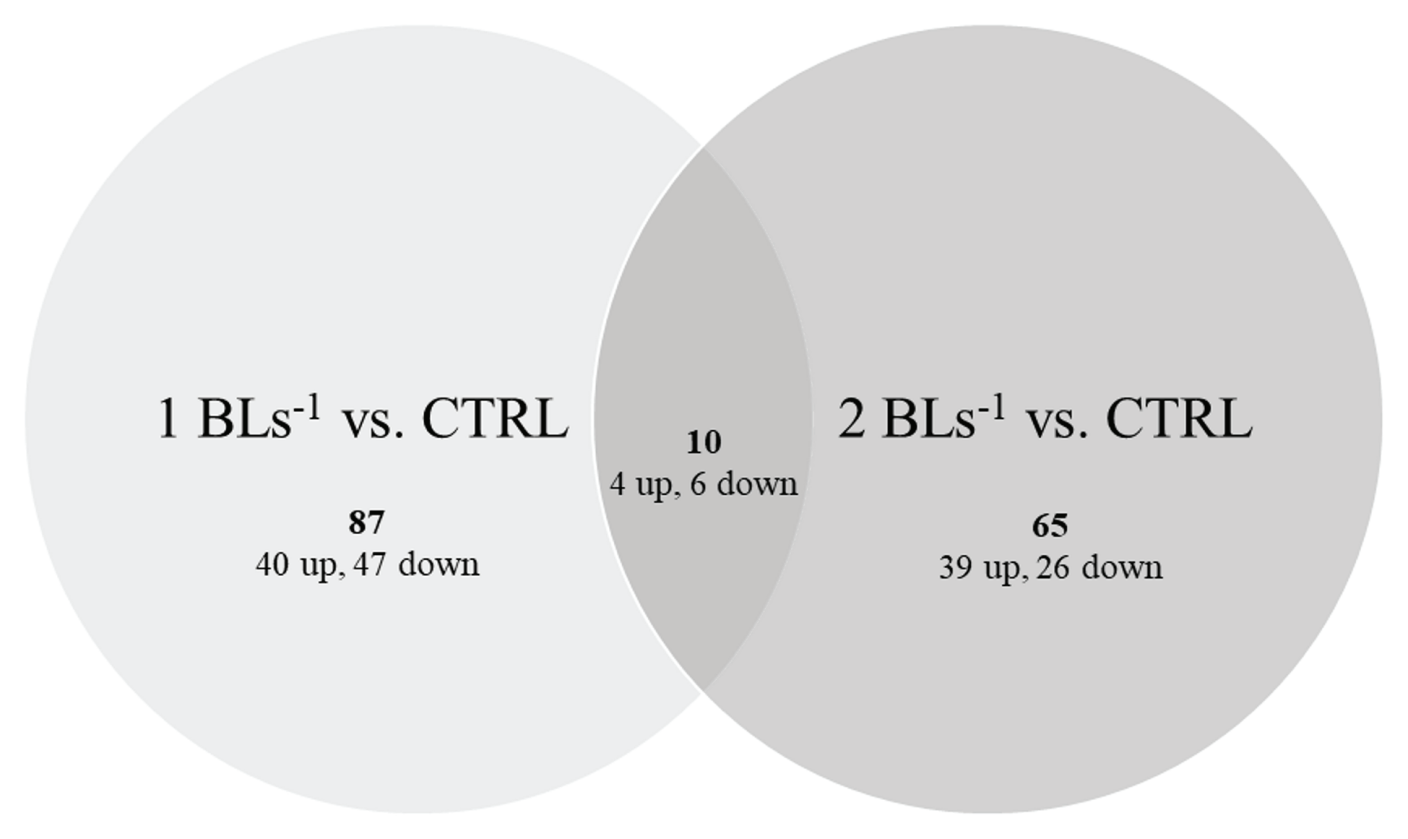
Differentially expressed genes (DEGs). Up- and down-regulated DEGs are shown in fish swimming at 1 BL s^−1^ (left) and 2 BL s^−1^ (right) vs. the controls with the overlap showing the common DEGs between both treatments.

Both treatments had only 10 DEGs in common (Figure 3; Table 3) of which four were upregulated and six were downregulated, too few to perform GO analysis on. Among the upregulated DEGS were Kelch41, MHC class II antigen beta (MHCII*β*), DM-related transcriptional factor 2b (DMRT2b), and angiopoietin-1. Kelch 41 was not expressed in the controls, and then upregulated in the flow conditioned fish in relation to exercise load.

**Table 3:** Common differentially expressed genes (DEGs).

Common DEGs	1 BL s^−1^ vs. C	2 BL s^−1^ vs. C
Kelch 41	Inf	Inf
MHC class II antigen beta partial	2.80	2.42
DM-related transcriptional factor Dmrt2b	2.52	2.83
Angiopoietin-1	2.06	2.15
Anoctamin-1-like isoform X2	0.56	0.55
Cordon-bleu isoform X5	0.55	0.56
Unconventional myosin-XVIIIa isoform X5	0.42	0.60
MTSS1 isoform X1	0.40	0.38
Transmembrane 145	0.13	0.14
Sodium channel type 4 subunit alpha B-like isoform X2	0.06	0.40

Common genes for the muscle transcriptome comparisons of fish swimming at 1 body length (BL) per second (1 BL s^−1^) vs. controls, and 2 BL s^−1^ vs. controls. Data are shown as fold change between each treatment group versus the control group. “Inf” refers to significant expression in the treatment groups but zero expression in the control group.

Thus, 87 DEGs (40 up and 47 down) were specific for flow conditioning at 1 BL s^−1^, 65 DEGs (39 up and 26 down) were specific for flow conditioning at 2 BL s^−1^ (Figure 3). GO of upregulated DEGs for flow conditioning at 1 BL s^−1^ is shown in Table 4. DEGs were functionally clustered as transcription regulators; as immune factors, and as involved in muscle contraction. Highly upregulated transcription regulators were three zinc fingers containing transcription factors and among them, as for the common DEGs, a *dmrt2* transcript. Immune-related genes included another transcription factor interferon regulatory factor 9 (*irf9*); C-C chemokine receptor type 7 (*ccr7*), and rab5 GDP GTP exchange factor (*rabgef1*)-like. The highly upregulated fibrinogen alpha chain (*fga*) is more involved in blood coagulation and chemotaxis. Troponin (*tn*) and two myosins (*myh* and *myl* gene) were the structural muscle genes involved in contraction, Prostaglandin F synthase (*prxl2b*) is an enzyme involved in the synthesis of PGF that stimulates contraction, and 5-AMP-activated kinase subunit gamma-1 (*prkag1*) is the energy sensor protein kinase indirectly responsible for metabolically fueling contraction.

**Table 4:** Gene ontology of upregulated DEGs for flow conditioning at 1 BL s^−1^.

	fc
**Regulation of transcription**	
NFX1-type zinc finger-containing 1	7.73
Double sex- and mab-3-related transcription factor 2	5.39
Zinc finger 804A	4.32
DNA-binding inhibitor ID-2	3.67
Pro-cathepsin H	2.88
5-AMP-activated kinase subunit gamma-1-like	2.61
**Immune system response; blood coagulation; chemotaxis**	
Fibrinogen alpha chain	17.51
C-C chemokine receptor type 7	13.96
Interferon regulatory factor 9	8.14
rab5 GDP GTP exchange factor-like	3.94
Pro-cathepsin H	2.88
ras-related C3 botulinum toxin substrate 2	2.65
HLA class II histocompatibility antigen gamma chain	1.83
**Muscle contraction**	
Troponin skeletal muscle	3.53
Prostaglandin F synthase-like	2.75
5-AMP-activated kinase subunit gamma-1-like	2.61
Myosin heavy partial	2.28
Myosin regulatory light chain skeletal muscle isoform-like	1.91

Data are shown as fold change between each treatment group vs. the control group.

Gene ontology of upregulated DEGs for flow conditioning at 2 BL s^−1^ is shown in Table 5. DEGs were functionally clustered again as transcription regulators and immune factors but also as involved in ossification, detection of calcium ion, skeletal system development, cartilage condensation, and chondrocyte development. Histone H2B plays an important role in the regulation of transcription as well as RNA polymerase II subunit A C-terminal domain phosphatase (*ssu72*) isoform X1, and zinc finger and SCAN domain-containing 22-like isoform X1 (*zscan22*). Upregulated immune-related genes included MHC class I (*mhcI*) and caspase-8 (*casp8*)-like and granzyme B-(*grb*)-like genes, which are involved in apoptosis. Several upregulated muscle genes were typical for skeletal development such as calsequestrin-2 (*casq2*)-like, collagen alpha-1(XI) chain (*col11a1*)-like isoform X1, hyaluronan and proteoglycan link 1 (*hapln1*), and extracellular calcium-sensing receptor (*car*)-like.

**Table 5:** Gene ontology of up-regulated DEGs for flow conditioning at 2 BL s^−1^.

	fc
**Regulation of transcription**	
Histone H2B	9.29
RNA polymerase II subunit A C-terminal domain phosphatase isoform X1	3.53
zinc finger and SCAN domain-containing 22-like isoform X1	2.77
Dual specificity phosphatase 22-A-like	1.82
HEXIM-like	1.80
Polyubiquitin-B	1.75
**Immune system process; proteolysis; apoptotic process**	
Caspase-8-like	7.86
Granzyme B-like	4.00
N-acetylated-alpha-linked acidic dipeptidase	3.38
MHC class I partial	Inf
**Muscle contraction**	
Calsequestrin-2-like	2.94
Collagen alpha-1(XI) chain-like isoform X1	2.13
Hyaluronan and proteoglycan link 1	1.64
Extracellular calcium-sensing receptor-like	Inf

Data are shown as fold change between each treatment group vs. the control group. “Inf” refers to significant expression in the treatment groups but zero expression in the control group.

## Discussion

This study reports on the physiological effects of continuous water flow-induced swimming exercise at speeds of 1 and 2 BL s^−1^ over a period of 8 months in seabream. Flow-induced exercise was expected to stimulate growth performance and robustness and resilience traits. Mechanistic insights were obtained in the role of the GH-IGF1 growth axis and the molecular regulation of white skeletal muscle development. It is important to note that effects of flow conditioning may be more than of muscle exercise alone. Additional effects may come from the increased water flow over the gills and therewith higher oxygen availability, and improvement of water quality in the tank by faster waste removal.

### Flow-Enhanced Growth and Physiological Adaptations

Increased water flow enhanced growth in relation with the applied swimming speed. Growth performance was also more uniform for fish under increased flow than for fish at minimal flow. Growth differences became consistent after 4 months of flow conditioning, when fish swimming at 2 BL s^−1^ reached 19.8 ± 6.7 g (40% higher than controls) and fish swimming at 1 BL s^−1^ reached 18.0 ± 6.2 g (28% higher than the controls) vs. the controls at 14.1 ± 4.5 g. Our increased growth percentages are high in comparison with an earlier report on exercised seabream fingerlings that weighed ~5 g at the start and were exercised at 5 BL s^−1^ for 5 weeks (Blasco et al., 2015). These exercised fingerlings reached an average weight of 20.3 ± 0.4 g and increased 16% more in body weight than the non-exercised controls that weighed 17.5 ± 0.5 g. Faster growth and smaller difference between exercised and control fingerlings was probably due to a difference in conditions between both studies, a water temperature of 23°C and a photoregime of 15L:9D (Blasco et al., 2015) vs. 18°C and a natural photoregime in our study. At the end of the experimental period in our study, fish body weight was 15% higher for fish swimming at 1 BL s^−1^ and 36% higher for fish swimming at 2 BL s^−1^. Similar to fingerlings, this percentage was high in comparison with an earlier study that reported 9% for seabream juveniles of ~90 g at the start, exercised at 1.5 BL s^−1^ for 1 month at 20°C and a photoregime of 12L:12D (Ibarz et al., 2011). Moya et al. (2019) recently showed that hyperplasia was responsible for exercise-enhanced muscle growth in fingerlings. Ibarz et al. (2011) showed that exercise-enhanced growth in juvenile seabream originated from muscle hypertrophy.

In earlier studies, we have shown that the carangid yellowtail kingfish showed major exercise-enhanced growth at their optimal metabolic swimming speeds. Exercised yellowtails increased their body weight by 46% as compared to resting fish after 18 experimental days while feeding them with equal rations (Palstra et al., 2015). The moronid European seabass (Graziano et al., 2018) and the sparid Gilthead seabream (Graziano and Palstra, unpublished data) did not show exercise-enhanced growth at their optimal swimming speed, but at much lower speeds (Ibarz et al., 2011). In this unpublished study, swimmers (*N* = 12) swam continuously for 24 days at 0.67 cm s^−1^ (3.48 SL s^−1^ which is optimal for fish of this size) and were then compared with resting fish (*N* = 12). Resting fish increased in body weight from 190 ± 10 to 225 ± 12 g while swimmers showed much less increase from 180 ± 8 to 192 ± 9 g. With our current study, we can confirm that enhanced growth for seabream occurs at lower than optimal metabolic swimming speeds, at speeds of 1 and 2 BL s^−1^.

Gilthead seabream is a benthic carnivore that needs to maneuver in the unsteady flows of the tides. Seabass is more an ambush hunter than pursuit hunter that catches its prey in a fast sprint. The musculature of both species is dominated by white muscle that matches the need for such feeding strategies. Both species do not adhere to hypothesis of Davison and Herbert (2013) for the strong relation between optimal metabolic swimming speed and optimal speed for growth. Still, seabream (Graziano and Palstra, unpublished results) and seabass (Graziano et al., 2018) can perform sustained swimming at optimal metabolic swimming speeds for months without fatigue. But, as we confirm in this study for seabream, the optimal speed for growth is lower than optimal metabolic swimming speed. In fact, sustained swimming regimes may not be the right strategy for growth enhancement in seabream, and seabass, and alternative flow regimes should be investigated. For example, flow can be applied only during a part of the day such as the 6 h exercise per day protocol for growth enhancement in zebrafish (Palstra et al., 2010, 2014). Exercised zebrafish increased their body weight by 17% as compared to resting fish after 20 experimental days (Palstra et al., 2019). Interval training may also represent an alternative exercise regime (Castro et al., 2011).

Flow-enhanced growth was accompanied by physiological adaptations to swimming exercise such as larger hearts and higher plasma glucose. Heart weights increase with exercise load as part of the transformation to a more aerobic phenotype as a response to sustained exercise. Increased cardiac output is required to provide the skeletal muscles and organs with blood and fuel and is a common response of fish to long term exercise (reviewed by Rodnick and Planas, 2016). Higher plasma glucose levels were found in the seabream conditioned at 1 BL s^−1^ while plasma glucose levels in fish conditioned at 2 BL s^−1^ were however similar to the levels in the controls. Plasma glucose levels in seabream that was exercised at 1.5 BL s^−1^ for 1 month did not significantly differ from the resting controls (Sánchez-Gurmaches et al., 2013). Glucose is thus important as fuel at lower, aerobically maintainable speeds of 1 BL s^−1^. Contrary to our expectation, mesenteric fat increased with flow, also when expressed as index relative to body weight (MFI). Faster growth is generally associated with less mesenteric fat and a leaner phenotype, also in seabream (Simó-Mirabet et al., 2018). Flow-enhanced lordotic fish may explain this observation (see section Flow-Enhanced Vertebral Lordosis).

### Flow-Enhanced Stress Resilience

Flow-conditioned seabream swimming at a speed of 1 BL s^−1^ had a lower baseline cortisol level than the controls, but also lower than the levels of the fish conditioned at 2 BL s^−1^. Exercise thus lowered the cortisol stress levels, but only at the lower exercise load, signifying eustress when swimming at 1 BL s^−1^ (Korte et al., 2005; Samaras et al., 2018). The absence of significant difference in cortisol levels between groups 30 and 90 min after the netting stressor showed that there were neither no consequences of flow-conditioning for peak magnitude nor for the recovery time to return to baseline, the two parameters that determine the resilience to the acute stressor. Cortisol levels peaked between 40 and 50 min after the stressor and highest levels measured were 52 ng ml^−1^. Peaking appeared to be 10–20 min later (Figure 2) than the 30 min as reported by Arends et al. (1999). Average and maximum levels, and individual variation, were not as high as reported by Castanheira et al. (2013) (36 ± 33 and 41 ± 28 ng ml^−1^ for average peaking with a range of 6–117 ng ml^−1^). It can be concluded that flow conditioning at 1 BL s^−1^ lowered stress but flow conditioning did not seem to increase stress resilience.

### Flow-Enhanced Vertebral Lordosis

Although flow conditioning enhanced growth performance, the percentage of vertebral lordosis dramatically increased when swimming at the highest speed of 2 BL s^−1^. Apparently, the mechanical load of exercise at this speed is too high for a developing juvenile. Exercise induces both muscle hyperplasia (Moya et al., 2019) and bone formation and mineralization (Suniaga et al., 2018), but myogenesis may occur at higher rate than skeletogenesis in relation to the exercise load. Vertebral lordosis also occurred in European seabass in response to water current (Divanach et al., 1997). At smaller size, a swimming speed of 2 BL s^−1^ may still not be a problem as Kihara et al. (2002) showed that swimming activity at 4, but not at 2 BL s^−1^, caused lordosis in juvenile red seabream *Pagrus major* of 25 mm. However, when growing larger, the swimming speed as defined in relation to body length may be too high and the absolute swimming speed should be maintained. Besides exercise load, one or more factors were involved in the induction of vertebral lordosis because the control group consisted of 13% lordotic fish in the absence of exercise. This percentage is in line with the 17% recently reported for hatchery reared seabream by Fragkoulis et al. (2019) who showed that seabream can recover from haemal lordosis during ongrowing in sea cages. Malfunction of the swim-bladder may cause lordosis but only affects prehaemal vertebrae in seabream and seabass (Chatain, 1994; Andrades et al., 1996). In seabream, the haemal lordosis may also develop due to early notochord abnormalities (Loizides et al., 2014). Vitamin A affects lordosis development in the later stages in seabass (Mazurais et al., 2009). Water temperature during the embryonic and larval phase affects the resistance/sensitivity of the later developmental stages (late metamorphosis and juvenile) to the swimming induced lordosis in European seabass (Sfakianakis et al., 2006). A combination of factor(s) and the 2 BL s^−1^ exercise regime may work synergistically in causing the morphological abnormalities. Although tank densities were maintained over time, fish size increased and, in combination with the 2 BL s^−1^ exercise regime in the same tank, may also have contributed to the occurrence of lordosis. The earlier reports on exercise-enhanced growth in seabream (e.g., Ibarz et al., 2011; Blasco et al., 2015) did not mention the occurrence of exercise-induced lordosis at similar low to moderate speeds. Recent research performed in zebrafish larvae confirms that exercise induction of lordosis follows a clear dose-response curve (Printzi et al., 2020).

Importantly, further data analysis of flow-enhanced lordotic fish showed significantly higher *K* (for fish of all three treatments) and MFI (for the exercised fish) values in comparison with normal formed fish (no differences for SL, BW, and CI). This observation shows that the unexpected higher MFI for exercised fish is due to an increased percentage of malformed fish. The fact that higher MFI only applies to exercised fish and not to the controls reflects an adaptation of malformed fish to exercise e.g., to obtain a more hydrodynamic shape and release the drag resistance.

### The GH-IGF1 Growth Axis

The absence of important differences in plasma GH, IGF1, and the IGF1/GH ratio (Pérez-Sánchez and Le Bail, 1999), and differential expression levels of the receptors in heart and white skeletal muscle, indicated no significant involvement of the growth axis in the flow-enhanced growth in this study. This unexpected but exciting finding is in contradiction with the existing literature that reports on major involvement of the GH-IGF1 growth axis in the exercise-enhanced growth in seabream. Sánchez-Gurmaches et al. (2013) showed that exercise-enhanced growth in seabream (~90 g at the start) is linked to increased plasma levels of IGF1 suggesting an important role for the GH-IGF1 growth axis. Five weeks of sustained exercise at 5 BL s^−1^ of seabream fingerlings (~5 g at the start) enhanced growth and caused plasma IGF1 to rise and plasma GH to reduce (Blasco et al., 2015). Vélez et al. (2016) reported on numerous modulations in the GH-IGF1 axis in seabream fingerlings such as a higher plasma IGF1/GH ratio, increased hepatic *igf1* expression and muscle *igf1c* and *ghr1* expression; increased muscle IGFBP5b and IGF2, and reduced hepatic IGF1Rb and both hepatic GHRs. If not the GH-IGF1 growth axis, what then played a major role in the exercise-enhanced growth as indicated on molecular regulatory level in this study?

### The Transcriptome of the Exercised Seabream Muscle and the Molecular Regulation of Muscle Building

Key muscle genes in flow-induced exercise (upregulated for both treatments) that show consistent differential expression are Kelch 41 (*klhl41*), involved in skeletal muscle development and differentiation (Gupta and Beggs, 2014); transcription factor *dmrt2b*; angiopoietin-1, involved in angiogenesis, and MHC class II antigen beta partial, involved in antigen processing and presentation (Table 3). Together with the DEGs that show strong upregulated expression for one of each of the flow treatments (Tables 3 and 4), they indicate that important changes occur in the processes of muscle contraction, muscle development, and its molecular regulation, and support the suggestion that immune genes play an important role in muscle repair and regeneration capacity.

RNAseq data in our study showed that transcription regulators play an important role in muscle growth. A transcription factor that showed upregulated expression for both flow treatments is *dmrt2b*. Dmrt genes are important developmental regulators (reviewed by Hong et al., 2007). In zebrafish larvae, Dmrt2 regulates muscle development and somite differentiation, and is involved in establishing left–right asymmetry (Meng et al., 1999; Saude et al., 2005; Lourenço et al., 2010; Matsui and Bessho, 2012). The specific role of Dmrt2b in juvenile seabream muscle is not yet known. Also, other transcriptional regulators may play an important role in exercise-enhanced muscle growth in seabream (*znfx1*; *znf804a*; *zscan22*; *ssu72*). Among them is H2B, a transcriptional regulator that was identified as an antimicrobial peptide in seabream as its expression is upregulated in response to an immune stimulus (Valero et al., 2016, 2020).

Expression of several immune genes was strongly upregulated, a common feature of the fish muscle under exercise (Palstra et al., 2013, 2014). Not only could the exercised muscle contribute to an improved immune capacity through the production of myokines (Pedersen et al., 2007), immune genes may also play an important role in muscle development. Exercise in mammals causes a (limited) acute inflammatory response to facilitate the repairing process for site-specific tissue damage (Shek and Shephard, 1998). Therewith, the immune genes involved promote skeletal and cardiac muscle training adaptation and regeneration (Tidball, 2017). Particularly in fish, exercise-induced muscle regeneration can be considerable (Rovira et al., 2018). RNAseq data of several exercised fish species reflect that a limited exercise-induced immune response occurs although functional studies are still lacking. Future studies should aim at elucidating the role of immune genes in muscle development of exercised fish.

Muscle of fish conditioned at both exercise regimes shows upregulated expression of structural genes involved in muscle contraction, calcium regulation, and the extracellular matrix. In muscle of fish conditioned at 1 BL s^−1^, expression of troponin, myosins, *prxl2b* and *prkag1* is upregulated. In muscle of fish conditioned at 2 BL s^−1^, these genes are extracellular calcium-sensing receptor-like, calsequestrin-2-like, collagen alpha-1(XI) chain-like isoform X1, and hyaluronan and proteoglycan link 1. Although only normally developed fish were selected for RNAseq, it may still be that particularly the latter two genes functionally link to muscle adaptations to lordosis and as such require more research attention.

### Conclusions and Perspectives for the Farmer

Flow-induced exercise is beneficial for growth (body weight increase) and uniformity (lower variation in body weight), stress (lower baseline plasma cortisol at 1 BL s^−1^), robustness (higher condition factor, larger hearts), energy mobilization (increased plasma glucose at 1 BL s^−1^), and muscle building (upregulated expression of genes involved in muscle contraction, muscle development and its molecular regulation, and immune genes). However, a serious constraint is the higher percentage of fish with vertebral lordosis in exercised fish, particularly at the highest flow condition of 2 BL s^−1^.

When an extension of the on-land period is considered, the optimal exercise regime for juvenile seabream would be at a flow inducing swimming exercise at 1 BL s^−1^. We suggest that a potentially limited increase of vertebral lordosis may be avoided by inducing exercise for only part of the day (6 h) or by starting flow conditioning at a later stage (~14 g) to avoid excessive exercise load at the earliest developmental stage. Exercise-enhanced growth of 15%, achieved by increased feed intake and/or lower feed conversion ratio, will shorten the production cycle. The more robust seabream are expected to show lower disease and mortality rates. Changes in the muscle structure of exercised seabream may reflect more “meaty” flesh and, together with hydrodynamic changes in body shape, create more resemblance to wild seabream which is important for the whole-fish market. Applied research to provide supporting evidence for these promising statements is recommended.

## Data Availability Statement

The datasets presented in this study can be found in online repositories. The names of the repository/repositories and accession number(s) can be found at: https://www.ncbi.nlm.nih.gov/geo/, GSE151668.

## Ethics Statement

The animal study was reviewed and approved by the Animal Experimental Commission of the Autonomous Government of Catalonia with protocol number 10323, and fish were always carefully handled by accredited staff.

## Author Contributions

AP and AR: conception and design of the study. AP, LK, PJ, JP-S, RD, and AR: acquisition of data, analysis and interpretation of data, and drafting and reviewing the manuscript. All authors contributed to the article and approved the submitted version.

### Conflict of Interest

Author RD was employed by the company Future Genomics Technologies B.V.

The remaining authors declare that the research was conducted in the absence of any commercial or financial relationships that could be construed as a potential conflict of interest.

## Footnotes

^1^https://www.ncbi.nlm.nih.gov/geo/query/acc.cgi?acc=GSE151668

## Abbreviations

BL: Body length
BL s^−1^: Body length per second
BW: Body weight
C: Control group
*car*: Extracellular calcium-sensing receptor
*casp8*: Caspase-8
*casq2*: Calsequestrin-2
*ccr7*: C-C chemokine receptor type 7
chol: Cholesterol
CI: Cardiac index
*col11a1*: Collagen alpha-1(XI) chain
DEGs: Differentially expressed genes
*dmrt2b*: DM-related transcriptional factor 2b
DO: Dissolved oxygen
*fga*: Fibrinogen alpha chain
GH: Growth hormone
*ghr1*: Growth hormone receptor 1
*ghr2*: Growth hormone receptor 2
gluc: Glucose
GO: Gene ontology
*grb*: Granzyme B
*hapln1*: Hyaluronan and proteoglycan link 1
Hct: Hematocrit
HSI: Hepatosomatic index
HW: Heart weight
I: Index
*igf1ra*: Insulin-like growth factor 1 receptor a
*igf1rb*: Insulin-like growth factor 1 receptor b
IGF1: Insulin-like growth factor 1
II: Intestinal index
*irf9*: Interferon regulatory factor 9
IW: Intestinal weight
K: Condition factor
klhl41: Kelch 41
lact: Lactate
LW: Liver weight
MFI: Mesenteric fat index
MFW: Mesenteric fat weight
*mhcI*: MHC class I
*MHCIIβ*: MHC class II antigen beta
*prkag1*: 5-AMP-activated kinase subunit gamma-1
*prxl2b*: Prostaglandin 5 synthase
*rabgef1*: rab5 GDP GTP exchange factor
RIA: Radiommunoassay
RNAseq: RNA sequencing
SI: Spleen index
SL: Standard lengh
SL s^−1^: Standard length per second
*ssu*: RNA polymerase II subunit A C-terminal domain phosphatase
SW: Spleen weight
tg: Triglyceride
*tn*: Troponin
W: Weight
*zscan22*: Zinc finger and SCAN domain-containing 22-like isoform X1
